# Prepregnancy Lifestyle Risk Factors in Women Seeking Digital Fertility Services: Cross-Sectional Descriptive Study

**DOI:** 10.2196/83023

**Published:** 2026-04-27

**Authors:** Jorge Chavarro, Audrey J Gaskins, Nara Lee, Elizabeth Murdoch, Peter N Schlegel, Tanvi Suresh

**Affiliations:** 1 Harvard T H Chan School of Public Health Boston, MA United States; 2 Rollins School of Public Health at Emory University Atlanta, GA United States; 3 Doveras Fertility Inc New York, NY United States; 4 New York Men’s Health Medical New York, NY United States

**Keywords:** prepregnancy lifestyle, digital health, maternal health, fertility, preconception care

## Abstract

**Background:**

Approximately 1 in 3 pregnancies in the United States are complicated by one or more adverse pregnancy outcomes. This high prevalence contributes to the elevated rates of maternal and infant mortality in the United States. Modifiable prepregnancy or preconception lifestyle factors have been associated with adverse pregnancy outcomes in observational studies, which underscores the importance of preconception care.

**Objective:**

This cross-sectional descriptive study aimed to (1) estimate the prevalence of preconception lifestyle risk factors among women seeking services from a digital fertility platform, (2) characterize the study population and present relevant reference data, and (3) examine the distribution of prepregnancy lifestyle scores across demographic and clinical subgroups.

**Methods:**

The digital health company, Doveras Fertility, has built a prepregnancy digital health platform for individuals and couples seeking to optimize their fertility potential. Targeting users prior to initiation of pregnancy, the platform facilitates the assessment of baseline lifestyle risk factors. This paper reports on 396 adult women who sought the platform’s services in a 1-month period between May and June 2024 by completing a digital fertility questionnaire. Self-reported data were analyzed for 6 healthy prepregnancy lifestyle factors known to be associated with maternal health outcomes in prior observational studies, and each participant was given a composite score between 0 to 6 to represent the number of these healthy behaviors reported. The 6 healthy prepregnancy lifestyle factors include a BMI of 18.5 to 24.9 kg/m^2^, not currently smoking, ≥150 min/week of moderate to vigorous physical activity, healthy eating, no daily alcohol intake, and use of a prenatal multivitamin.

**Results:**

The study population was racially and ethnically diverse, with a mean age of 32.9 (SD 6.3) years. Most (235/396, 59%) participants received a composite score of 3 factors or fewer, and less than 5% (19/396) scored 6 out of 6. For context, this cohort had higher proportions of participants with unhealthy BMI and dietary patterns than those in the reference data. Regarding fertility, 46% (182/396) met the clinical definition of infertility (≥1 year trying to conceive), with the prevalence of infertility ranging from 16% (3/19) among those with the highest lifestyle scores to 59% (17/29) among those with the lowest.

**Conclusions:**

Most women seeking services from this digital fertility platform exhibited multiple lifestyle factors that have been previously associated with adverse pregnancy outcomes in observational studies. These results suggest that nearly all survey participants have potential risk factors for adverse maternal outcomes and therefore the potential to adopt at least one improvement in their lifestyle behavior. A digital platform may offer an accessible mechanism for identifying and characterizing preconception risk factors; however, future longitudinal studies are needed to evaluate whether platform-based interventions can effectively support behavior change and improve maternal health outcomes.

## Introduction

The United States has witnessed a critically worsening maternal mortality rate—one that is several times higher than that of other industrialized and high-income countries and adversely affects the overall quality of health care in the United States [1]. The real-world experience for the American woman is that she is more than twice as likely to die from complications related to pregnancy or childbirth than a woman in another high-income nation [1]. Intervention during the prepregnancy period may help reduce not only the incidence of maternal health risks but also the associated costs for payers: every US $1 invested in prepregnancy care may save up to US $5 on infant and maternal health costs [2]. There are multiple approaches that may reverse the worsening maternal mortality rate in the United States and improve maternal health outcomes overall [3].

Evidence supporting the impact of preconception lifestyle factors continues to grow. For example, the Nurses’ Health Study II, a large longitudinal cohort study run by Harvard Medical School, Brigham and Women’s Hospital, and Harvard TH Chan School of Public Health, showed that women with the highest adherence to a *prepregnancy healthy lifestyle* had a substantially lower risk of adverse pregnancy outcomes and suggested that these lifestyle factors could be targeted as an effective intervention for the prevention of adverse pregnancy outcomes [4]. The benefits of a prepregnancy healthy lifestyle extend beyond the mother to the infants as well. A study from the Pregnancy Environment and Lifestyle Study cohort found that a healthy diet, healthy weight, and low levels of stress during the preconception period were associated with reduced risk of preterm birth [5], and a meta-analysis of 17 clinical trials found that micronutrient supplementation during the preconception period reduced the risk of having an infant with low birth weight [6]. However, more intervention studies are needed to effectively demonstrate that lifestyle interventions during this critical preconception period can significantly reduce both maternal and infant health risks.

Given the high-quality evidence for the importance of lifestyle factors in the preconception window for maternal health outcomes, numerous public health bodies encourage women trying to conceive to engage in preconception counseling. According to the American College of Obstetricians and Gynecologists, “the goal of prepregnancy care is to reduce the risk of adverse health effects by optimizing health and addressing modifiable risk factors” [7]. The World Health Organization has emphasized preconception care as a public health priority, and the International Federation of Gynecology and Obstetrics (FIGO) has released comprehensive recommendations highlighting the importance of optimizing maternal health before conception [8]. Despite this recognition, significant barriers to preconception care delivery persist, including lack of awareness among both patients and clinicians, socioeconomic factors limiting health care access, and competing health care priorities [9,10].

Given these barriers, it is likely that the opportunity for preconception intervention to reduce adverse maternal health risks is not being well captured by the current health system [10-13]. More effective and widespread intervention might be possible through a digital health solution. By capturing those at the high engagement point of trying to conceive, interventions might be able to target people in the important preconception window to assess a user’s potential risks and deliver personalized intervention in an accessible and affordable way.

The aim of this cross-sectional descriptive study was to (1) estimate the prevalence of preconception lifestyle risk factors among women seeking services from a digital fertility platform, (2) characterize the study population and present relevant reference data, and (3) examine the distribution of prepregnancy lifestyle scores across demographic and clinical subgroups. We hypothesized that this population would exhibit modifiable lifestyle factors that have been previously associated with adverse pregnancy outcomes in observational studies.

## Methods

### Study Design and Setting

This was a cross-sectional descriptive study analyzing anonymized data from individuals who sought services from the Doveras digital health platform. Doveras has designed and developed preconception lifestyle and behavioral intervention programs, delivered through its digital health platform, for individuals and couples to improve various end points along the reproductive health journey. These personalized interventions are based on relevant data from existing clinical studies on preconception lifestyle factors and their impacts on various reproductive health end points. The platform (1) facilitates the assessment of baseline lifestyle risk factors, (2) identifies modifiable behavior opportunities and potential adverse fertility and maternal health risks to mitigate, and (3) delivers personalized programming to help users sustain simple behavior changes that target their unique risk to improve their overall reproductive health outcomes.

### Participants

This study evaluated the anonymized data of nearly 400 verified participants who sought services from the Doveras digital health platform during a 1-month period (mid-May to mid-June 2024). These participants were directed to the Doveras website through their engagement with social media advertisements (including platforms such as Facebook and Instagram) to improve fertility potential with personalized, data-driven lifestyle interventions. Enrollment criteria entailed completion of a digital fertility questionnaire encompassing self-reported personal demographics and various lifestyle and behavioral characteristics. The included participants were aged 18 years or older, who self-reported as female, and were attempting to conceive via sexual intercourse with a male partner. Individuals who reported they were using or planning to use assisted reproduction technologies to conceive were excluded. This exclusion was applied because participants using assisted reproductive technologies would likely receive specialized medical care that could confound assessment of lifestyle risks. During the enrollment period, 441 individuals completed the questionnaire. Of these, 45 (10.2%) were excluded for not meeting eligibility criteria (eg, n=41, 9.3% not attempting to conceive via sexual intercourse or n=4, 1% incomplete data), yielding 396 (89.8%) eligible participants included in the analysis.

### Data Collection and Measures

For the purposes of this study, self-reported behavioral and demographic data from these participants were analyzed using the maternal health risk framework developed through the Nurses’ Health Study II’s definition of a participant’s healthy prepregnancy lifestyle factor score [4]. This framework observed 15,509 women and 27,135 pregnancies and identified 6 healthy prepregnancy lifestyle factors associated with a lower risk of adverse maternal health outcomes. These factors included (1) healthy BMI of 18.5 to 24.9 kg/m^2^, (2) not currently smoking (cigarettes or e-cigarettes), (3) 150 min/wk or more of moderate to vigorous physical activity, (4) healthy eating, (5) <15 g/d alcohol, and (6) use of a prenatal multivitamin.

Participants answered questions on these and other topics as part of a single time point survey. Following the method from the Nurses’ Health Study II, self-reported behaviors of this study cohort were analyzed according to each of these 6 variables. These 6 variables were coded as binary (0 or 1) and summed for each participant to compute their total healthy prepregnancy lifestyle score. Regarding diet, participants who reported “I would say I mostly eat healthy” received a score of “1,” while those who answered, “I have some healthy habits” or “I would say my diet needs a lot of help to be considered healthy” received a score of “0.” For the smoking variable, participants who reported that they were a “social smoker” received a “1” as not regular smokers. For the alcohol variable, those who reported “no” to the question of whether they drink daily received a “1,” and those who responded “yes—1 drink daily” or “yes—2+ drinks daily” received a score of “0.” Participants also self-reported data on reproductive and clinical history.

### Statistical Analysis

Descriptive statistics are presented as means with SDs for normally distributed continuous variables, medians with IQRs for nonnormally distributed variables, and frequencies with percentages for categorical variables. A 1-way ANOVA was used to test for differences in mean values of continuous variables across the 6 groups of healthy lifestyle scores, while a chi-square test (or a Fisher test for low cell count values) was used to assess the association between categorical variables and these 6 groups. Effect sizes are reported as η² (eta-squared) for continuous variables and Cramér V for categorical variables. A *P* value of <.05 was considered statistically significant. All analyses were conducted using R (version 4.4.0, R Foundation for Statistical Computing). Missing data were minimal (<1% for any single variable); analyses included only participants with complete data for all variables. No formal sample size calculation was performed, as this was a descriptive study designed to characterize the population seeking services from the platform during the study period; all eligible participants were included.

### Ethical Considerations

The study met the criteria for human subject research exemption as determined by the Salus Institutional Review Board (protocol number 24072). The exemption was granted because the research involved the analysis of existing, anonymized data collected during routine platform operations under a Terms of Use and a privacy policy that disclosed the use of deidentified data for research. All data were deidentified prior to analysis, with no direct identifiers included in the research dataset. No monetary compensation was provided for questionnaire completion. Participants were offered a promotional discount for the platform’s services, consistent with standard onboarding procedures for all new users at that time.

## Results

### Participant Characteristics

The analysis included 396 female-identifying participants, with a mean age of 32.9 (SD 6.3) years and 50% (198/396) of participants identified as non-Hispanic White.

### Assessment of Healthy Prepregnancy Lifestyle Factors

Among survey participants, the median number of the 6 healthy prepregnancy lifestyle factors was 3.0 (IQR 2.0-4.0), with 59% (235/396) scoring 3 or below and 86% (340/396) scoring 4 or below (Table 1).

**Table 1:** Distribution of healthy prepregnancy lifestyle scores among survey participants (N=396).

Healthy prepregnancy lifestyle score	Participants, n (%)
0 or 1	29 (7.3)
2	83 (21)
3	123 (31.1)
4	105 (26.5)
5	37 (9.3)
6	19 (4.8)

The average participant had a median BMI of 31.9 (IQR 26.5-38.0), with 82% (325/396) of participants outside of a BMI range defined as healthy (18.5-24.9 kg/m^2^). There were significant differences in the mean BMI of participants across the different healthy prepregnancy lifestyle scores (*F*_5,390_=17.6; *P*<.001; η²=0.18), demonstrating that participants with lower healthy prepregnancy lifestyle scores had higher average BMI levels. Using the Nurses’ Health Study II and nationally representative samples from the National Health and Nutrition Examination Survey (NHANES) as references [4], the Doveras cohort had a greater proportion of respondents with unhealthy BMI and unhealthy diets (Table 2). However, the Doveras cohort’s smoking, physical activity, and alcohol intake were similar to those of these populations. These comparisons should be interpreted as contextual only, given differences in populations, periods, and measurement approaches across the studies.

**Table 2:** Presence of healthy prepregnancy lifestyle factors in Doveras cohort (2024), Nurses’ Health Study II (2005), and from nationally representative samples of women aged 18 to 44 years (National Health and Nutrition Examination Survey, NHANES; 2017-2018).

Healthy lifestyle factors	Doveras (2024; %)	Nurses’ Health Study II (2005; %)	NHANES (2017-2018; %)
BMI (18.5-24.9 kg/m^2^)	17.7	52.1	32.2
Current nonsmoker	88.4	94.3	81.4
Moderate to vigorous exercise (≥150 min/wk)	51.0	33.9	54.3
Healthy diet	21.2	38.6	40.0
Alcohol intake (0-15 g/d)	92.7	93.5	92.9
Multivitamin use	55.8	48.4	19.4

A total of 5% (19/396) of participants received the highest healthy prepregnancy lifestyle score of 6. Within this group, most participants were White (15/19, 79%).

Unsurprisingly, regarding behavioral characteristics, it was generally observed that participants with lower healthy prepregnancy lifestyle scores had lower proportions of healthy behavior. For example, among participants who said they rarely or never exercised (n=63), 83% (52/63) had a healthy prepregnancy lifestyle score of 3 or lower. As for diet, 25% (100/396) of participants felt their diet “needs a lot of work to be considered healthy.” In a similar pattern, 97% (109/112) of people who scored 2 or lower (n=112) believe they could use help with their diet. Furthermore, 44% (175/396) of all participants did not report taking prenatal vitamins.

However, there were certain healthy behaviors that most of the respondents reported. For example, 92% (367/396) of participants did not report daily alcohol use and 77% (306/396) did not report currently smoking. Although among those who did report drinking one or more drinks daily, 55% (16/29) had a healthy prepregnancy lifestyle score of 2 or lower suggesting that individuals who drink daily are more likely to lack other healthy lifestyle behaviors as well.

### Associated Health History

The survey also assessed self-reported past medical and fertility history (Table 3). Among 396 survey participants, 61 (15%) reported they had been diagnosed with prediabetes or diabetes, and 98 (25%) reported a close family member had been diagnosed with diabetes. Among participants who reported having diabetes or prediabetes (n=61), only 1 (2%) had a healthy prepregnancy lifestyle score of 5 or 6, suggesting individuals with these health conditions are at higher risk for adverse maternal health outcomes and would benefit from improvements in healthy behaviors.

**Table 3:** Participant characteristics by healthy prepregnancy lifestyle score.

			Healthy prepregnancy lifestyle score							*P* value		Effect size (V)^a^
			0 or 1	2	3	4	5	6				
Participants, n			29	83	123	105	37	19				
**Demographic characteristics**												
	**Race or ethnicity, n (%)**									.01		0.09
		American Indian or Alaskan native	0 (0)	2 (2.4)	2 (1.6)	1 (1)	0 (0)	0 (0)				
		Asian	0 (0)	2 (2.4)	5 (4)	6 (5.7)	2 (5.4)	1 (5.3)				
		Black or African American	11 (38)	31 (37)	23 (19)	23 (22)	2 (5.4)	0 (0)				
		Hispanic or Latino	2 (6.9)	11 (13)	16 (13)	14 (13)	7 (19)	1 (5.3)				
		Mixed Race	1 (3.4)	6 (7.2)	4 (3.3)	5 (4.8)	1 (2.7)	1 (5.3)				
		Prefer not to say	3 (10)	0 (0)	5 (4.1)	3 (2.9)	1 (2.7)	0 (0)				
		Race not listed	0 (0)	0 (0)	1 (0.8)	3 (2.9)	1 (2.7)	1 (5.2)				
		White	12 (41)	31 (37)	67 (55)	50 (48)	23 (62)	15 (79)				
	Age (years), mean (SD)		32.2 (6.5)	33.6 (6.5)	32.4 (6.3)	32.8 (6.3)	32.6 (5.6)	34.8 (5.7)	.55			
**Healthy prepregnancy lifestyle factors**												
	BMI, median (IQR)		33.2 (30.0-41.8)	35.3 (29.7-41.7)	33.4 (26.9-40.5)	30.0 (24.5-36.5)	25.4 (22.3-31.4)	21.0 (20.2-22.2)	<.001			
	**Smoking, n (%)**									<.001		0.32
		Yes	18 (62)	18 (22)	6 (4.9)	4 (3.8)	0 (0)	0 (0)				
		Social smoker	7 (24)	5 (6)	4 (3.3)	0 (0)	0 (0)	0 (0)				
		Former	1 (3.4)	7 (8.4)	3 (2.4)	14 (13)	2 (5.4)	1 (5.3)				
		No	3 (10)	53 (64)	110 (89)	87 (83)	35 (95)	18 (95)				
	**Physical activity, n (%)**									<.001		0.26
		I rarely or never work out	15 (52)	16 (19)	21 (28)	10 (9.5)	1 (2.7)	0 (0)				
		Approximately 0.5 h/wk	5 (17)	19 (23)	7 (5.7)	8 (7.6)	0 (0)	0 (0)				
		Approximately 1 h/wk	3 (10)	9 (11)	12 (9.8)	1 (0.95)	1 (2.7)	0 (0)				
		Approximately 1.5 h/wk	3 (10)	8 (9.6)	9 (7.3)	5 (4.8)	2 (5.4)	0 (0)				
		Approximately 2 h/wk	3 (10)	18 (22)	13 (11)	5 (4.8)	0 (0)	0 (0)				
		Approximately 2.5 h/wk	0 (0)	2 (2.4)	5 (4.1)	9 (8.6)	4 (11)	3 (16)				
		>3 h/wk	0 (0)	11 (13)	56 (46)	67 (64)	29 (78)	16 (84)				
	**Alcohol use, n (%)**									.63		0.11
		No	22 (76)	74 (89)	116 (94)	100 (95)	36 (97)	19 (100)				
		Yes—1 drink daily	5 (17)	7 (8.4)	5 (4)	5 (4.7)	1 (2.7)	0 (0)				
		Yes—>2 drinks daily	2 (6.8)	2 (2.4)	2 (1.6)	0 (0)	0 (0)	0 (0)				
	**Diet, n (%)**									<.001		0.50
		I would say I mostly eat healthy	0 (0)	3 (3.6)	4 (3.3)	29 (28)	29 (89)	19 (100)				
		I have some healthy habits	15 (52)	48 (58)	83 (67)	59 (56)	7 (19)	0 (0)				
		My diet needs a lot of work to be considered healthy	14 (48)	32 (39)	36 (29)	17 (16)	1 (2.7)	0 (0)				
	**Prenatal vitamin, n (%)**									<.001		0.62
		No	28 (97)	70 (84)	57 (46)	17 (16)	3 (8.1)	0 (0)				
		Yes	1 (3.4)	13 (16)	66 (54)	88 (84)	34 (92)	19 (100)				
**Reproductive and clinical history**												
	Clinical definition of infertility^b^, n (%)		17 (59)	39 (47)	65 (53)	45 (43)	13 (35)	3 (16)	.04		0.16	
	Previous birth, n (%)		7 (24)	36 (43)	59 (48)	51 (49)	11 (30)	3 (16)	.01		0.18	
	Hormone imbalance^c^, n (%)		20 (69)	57 (69)	78 (63)	69 (66)	28 (76)	16 (84)	.37		0.10	
	Diabetes or prediabetes, n (%)		9 (31)	12 (15)	17 (14)	22 (21)	1 (2.7)	0 (0)	.01		0.18	
	Endometriosis, n (%)		6 (21)	7 (8.4)	12 (9.8)	13 (13)	2 (5.4)	2 (11)	.51		0.11	

^a^η² for continuous variables are: <0.01 for age, 0.18 for BMI.

^b^Defined as self-reported duration of attempting to conceive for 12 months or longer.

^c^Self-reported diagnosis of any hormonal condition (eg, polycystic ovary syndrome, thyroid disorder, and luteal phase defect).

Regarding fertility, 46% (182/396) met the clinical definition of infertility (≥1 year trying to conceive), with the prevalence of infertility ranging from 16% (3/19) among those with the highest lifestyle scores to 59% (17/29) among those with the lowest. This high proportion of participants meeting criteria for infertility is notable given that individuals using or planning to use assisted reproductive technologies were excluded; this suggests the cohort may represent individuals in the intermediary period: those who have been trying to conceive for more than a year but have not yet initiated or decided to pursue assisted reproductive technology. Among those who reported giving birth prior to the program (n=167), 56% (10/18) of individuals with a prepregnancy healthy lifestyle score of 0 or 1 (n=18) took >1 year to conceive. In contrast, 15% (2/13) of those with a score of 6 (n=13) took the same amount of time to conceive.

## Discussion

### Principal Findings

This cross-sectional descriptive study characterized the prevalence of preconception lifestyle risk factors among 396 women seeking services from a digital fertility platform. The principal findings were that (1) most (n=235, 59%) of participants received a composite score of 3 factors or fewer and less than 5% scored 6 out of 6, (2) for context, this cohort had higher proportions of participants with unhealthy BMI and dietary patterns than those in the reference data, and (3) regarding fertility, 46% (n=182) met the clinical definition of infertility (≥1 year trying to conceive), with prevalence of infertility ranging from 16% (3/19) among those with the highest lifestyle scores to 59% (17/29) among those with the lowest.

### Comparison With Prior Work

#### Overview

The population of people seeking Doveras’ services presented, before joining the platform, with considerably lower healthy prepregnancy lifestyle factor scores than those reported in the Nurses’ Health Study. Since the Doveras preconception platform is marketed for its fertility benefits, this research may have oversampled individuals who are having trouble conceiving, which could contribute to why this cohort represents a particularly at-risk group for adverse pregnancy outcomes. The Doveras cohort is also demographically diverse, roughly matching the general population in racial and ethnic composition, though not necessarily in other characteristics, such as socioeconomic status or geographical distribution, which were not assessed. The unique lifestyle and demographic baseline of this cohort point to a few avenues of significance and areas for future research, discussed subsequently.

#### Prevalence of Modifiable Risk Factors

These results suggest that most people seeking preconception services during this period from Doveras have the potential to adopt additional healthy behaviors that have been associated with lower risk of adverse pregnancy outcomes in observational studies.

The lifestyle factor gaps exhibited in these participants during the preconception window have been shown to be associated with various adverse pregnancy outcomes in prior observational research. For example, a prospective cohort of 2449 women as part of the Pregnancy Environment and Lifestyle Study found that modifiable lifestyle factors such as healthy weight, high-quality diet, and low levels of stress were associated with a reduced risk of preterm birth by 42%, 32%, and 40%, respectively [5]. Additionally, a meta-analysis of 17 trials encompassing 137,791 women found that taking multiple micronutrient supplements resulted in reduced risk of having a newborn infant with low birth weight [6]. The Nurses’ Health Study II, which used the same 6 healthy prepregnancy lifestyle factors as this study, shows similar associations between prepregnancy adherence to these lifestyle factors and adverse outcomes. This research reported that approximately 1 in 3 pregnancies (9702/27,135, 36%) in the cohort were complicated by one or more adverse pregnancy outcomes. The researchers found that these 6 specific low-risk factors were inversely associated with risk of adverse pregnancy outcomes—including miscarriage, gestational hypertension, preeclampsia, gestational diabetes, preterm birth, and low birthweight—in a dose-dependent manner. Compared with women who had 0 or 1 healthy prepregnancy lifestyle factor, those with 6 had a 37% lower risk of adverse pregnancy outcomes, driven primarily by lower risks of gestational diabetes, gestational hypertension, and low birth weight.

A digital health platform may offer a mechanism for identifying these lifestyle risk factors and characterizing the preconception health status of individuals seeking fertility services. The Nurses’ Health Study researchers concluded that if the observed relationships between the studied behaviors and adverse maternal health outcomes were causal, 19% of adverse pregnancy outcomes could have been prevented by the adoption of all 6 healthy prepregnancy lifestyle factors [4]. However, as that study and this study are observational, causality cannot be established, and intervention studies are needed to determine whether promoting adoption of these factors will in fact reduce adverse outcomes.

Given that the Doveras cohort presents with lower healthy lifestyle (ie, less healthy) scores than the Nurses’ Health Study cohort, this population may represent a group with particularly high prevalence of modifiable risk factors. If the associations observed in prior research are causal, interventions targeting this population could potentially have meaningful effects.

#### Lifestyle Factors With Highest Prevalence of Unhealthy Patterns

This descriptive analysis also highlights which lifestyle factors showed the highest prevalence of unhealthy patterns in this cohort. Specifically, the Doveras population overindexed on the lack of these specific healthy factors: BMI, healthy diet, physical activity, and taking a prenatal vitamin, in that order. Among the Doveras participants, 82% (325/396) reported a high BMI (≥25 kg/m^2^), which has been associated with gestational hypertension, preeclampsia, gestational diabetes, and miscarriage in data from the Nurses’ Health Study II [4]. Similarly, 44% (175/396) of the study participants reported that they were not currently taking prenatal vitamins, which has been associated with higher rates of miscarriage and having infants with low birth weight [4].

The Nurses’ Health Study II also reported that the prepregnancy healthy lifestyle factors screened for, except avoiding harmful alcohol consumption and regular physical activity, were independently associated with lower risk of adverse pregnancy outcomes after mutual adjustment for each other. Healthy BMI, high-quality diet, and multivitamin supplementation showed the strongest inverse associations with adverse pregnancy outcomes.

Given the high prevalence of elevated BMI and suboptimal dietary patterns in this cohort, these lifestyle factors may represent particularly relevant targets for future intervention development and testing.

#### Digital Health Platforms for Preconception Care

A critical question is whether a digital intervention can facilitate sustained behavior change, and if so, whether such an intervention can decrease adverse pregnancy outcomes. Previous studies have suggested that lifestyle interventions deployed electronically during the preconception period can be feasible and may promote short-term behavior change [14-16]. A 2023 systematic review of mobile phone apps for preconception behavior change found growing interest in this area, although the limited number of studies and heterogeneity in outcomes precluded firm conclusions about effectiveness [15]. Smith et al [16] showed that nudging behavior change during prepregnancy digital interventions to encourage pregnancy preparation is feasible. These studies show the feasibility of digital platforms for delivering preconception health information and behavior change support. Future studies should validate the platform’s usability and behavior change capabilities.

### Limitations

This study has several important limitations. The cross-sectional descriptive design precludes causal inference; we only report associations and prevalence estimates.

The self-selection of the program is an inherent limitation that limits the generalizability. The Doveras cohort may not be fully representative of all individuals trying to conceive, as participants who opt into this product might be more likely to be experiencing fertility challenges or have a higher motivation to improve their lifestyle behaviors. Indeed, 46% (182/396) of participants met the clinical definition of infertility, despite the exclusion of those using or planning to use assisted reproductive technologies. This high proportion suggests the cohort may overrepresent individuals experiencing fertility challenges. Participants were recruited through social media advertisements for a commercial platform, which may select for individuals with internet access, engagement with fertility-related content, and motivation to improve fertility. The study did not include LGBTQ+ (lesbian, gay, bisexual, transgender, and queer) individuals, single women attempting to conceive through donor insemination, or those using assisted reproductive technologies, limiting applicability to these populations. Information on geographical location, income, and other socioeconomic factors was not studied, precluding assessment of representativeness along these dimensions.

Several measurement limitations should be noted. Key constructs such as diet quality, smoking status, and alcohol intake were measured using single self-reported items with dichotomized definitions that deviate from validated instruments used in prior studies. For example, our diet assessment relied on a single question about perceived diet quality rather than validated dietary assessment tools such as food frequency questionnaires. Future studies should incorporate validated instruments for key constructs such as diet quality and physical activity. Furthermore, as with all self-reported data, the potential for recall error is another concern.

Finally, comparisons with the Nurses’ Health Study II and NHANES should be interpreted with caution. These comparisons are indirect and unadjusted, involving different populations, periods, recruitment methods, and measurement tools. These comparisons are provided for contextual purposes only and do not represent direct statistical comparisons.

### Conclusions

This cross-sectional descriptive study found that most women seeking services from a digital fertility platform exhibited multiple lifestyle factors that have been previously associated with adverse pregnancy outcomes in observational studies, including high BMI (325/396, 82% outside healthy range), suboptimal dietary patterns (312/396, 79%), and lack of prenatal vitamin use (175/396, 44%). At least 86% (340/396) of people seeking fertility services from the Doveras digital health platform exhibited 2 or more known lifestyle risk factors that have been associated with adverse maternal health outcomes in prior research.

These findings suggest that a digital platform may offer an accessible mechanism for identifying and characterizing preconception risk factors among individuals seeking fertility services. Future longitudinal and intervention studies are needed to determine whether digital platform–based preconception interventions can effectively support sustained lifestyle modification and, ultimately, reduce adverse pregnancy outcomes.

## Data Availability

The datasets generated and analyzed during this study are not publicly available due to privacy considerations and proprietary platform data restrictions but are available from the corresponding author on reasonable request and with appropriate data use agreements.

## Abbreviations

FIGO: International Federation of Gynecology and Obstetrics
LGBTQ+: lesbian, gay, bisexual, transgender, and queer
NHANES: National Health and Nutrition Examination Survey
